# [^18^F]FDG PET accurately differentiates infected and non-infected non-unions after fracture fixation

**DOI:** 10.1007/s00259-016-3528-9

**Published:** 2016-10-05

**Authors:** Vera Wenter, Nathalie L. Albert, Matthias Brendel, Wolfgang P. Fendler, Clemens C. Cyran, Peter Bartenstein, Jan Friederichs, Jan-Philipp Müller, Matthias Militz, Marcus Hacker, Sven Hungerer

**Affiliations:** 1 Department of Nuclear Medicine, University of Munich, Munich, Germany; 2 Institute for Clinical Radiology, University of Munich, Munich, Germany; 3 Department of Reconstructive Arthroplasty, BG Trauma Center Murnau, Murnau, Germany; 4 Paracelsus Medical University, PMU Salzburg, Salzburg, Austria; 5 Division of Nuclear Medicine, Department of Biomedical Imaging and Image-guided Therapy, University of Vienna, Währinger Gürtel 18-20, Floor 5L, 1090 Vienna, Austria

**Keywords:** Non-union, Fractures, Infection, FDG, PET

## Abstract

**Purpose:**

Complete fracture healing is crucial for good patient outcomes. A major complication in the treatment of fractures is non-union. The pathogenesis of non-unions is not always clear, although implant-associated infections play a significant role, especially after surgical treatment of open fractures. We aimed to evaluate the value of [^18^F]FDG PET in suspected infections of non-union fractures.

**Methods:**

We retrospectively evaluated 35 consecutive patients seen between 2000 and 2015 with suspected infection of non-union fractures, treated at a level I trauma center. The patients underwent either [^18^F]FDG PET/CT (*N* = 24), [^18^F]FDG PET (*N* = 11) plus additional CT (*N* = 8), or conventional X-ray (*N* = 3). Imaging findings were correlated with final diagnosis based on intraoperative culture or follow-up.

**Results:**

In 13 of 35 patients (37 %), infection was proven by either positive intraoperative tissue culture (*N* = 12) or positive follow-up (*N* = 1). [^18^F]FDG PET revealed 11 true-positive, 19 true-negative, three false-positive, and two false-negative results, indicating sensitivity, specificity, positive predictive value (PPV), negative predictive value (NPV), and accuracy of 85 %, 86 %, 79 %, 90 %, and 86 %, respectively. The SUV_max_ was 6.4 ± 2.7 in the clinically infected group and 3.0 ± 1.7 in the clinically non-infected group (*p* <0.01). The SUV_ratio_ was 5.3 ± 3.3 in the clinically infected group and 2.6 ± 1.5 in the clinically non-infected group (*p* <0.01).

**Conclusion:**

[^18^F]FDG PET differentiates infected from non-infected non-unions with high accuracy in patients with suspected infections of non-union fractures, for whom other clinical findings were inconclusive for a local infection. [^18^F]FDG PET should be considered for therapeutic management of non-unions.

## Introduction

Approximately 5–10 % of fractures fail to heal properly, qualifying as delayed union or persistent non-union fractures [1]. Non-unions are radiologically classified as atrophic, oligotrophic, or hypertrophic, based in part on the particular pathogenesis of the non-union, which thus has an impact on further treatment options [2]. The occurrence of infection at the site of non-union is a major issue in selecting appropriate treatment; infection is considered the worst prognostic factor in tibia fracture care [3]. When stable fracture fixation can be confirmed, the presence of a bacterial infection is a possible reason for the persistence of a non-union [4]. The treatment algorithm in such cases becomes more radical and aggressive debridement, with installation of an external fixator.

The presence of an acute infection may be obvious due to open draining wounds, erythema, or incomplete soft tissue coverage. However, swelling, erythema, and hyperthermia can be suggestive of an infection, while merely indicating instability and local inflammatory reaction. Furthermore, a bacterial infection at the site of non-union can be subacute or chronic, and consequently more difficult to diagnose. Besides the clinical aspects, the diagnosis of infection is supported by laboratory blood tests such as C-reactive protein (CRP), erythrocyte sedimentation rate (ESR), and peripheral leukocyte count. However, these laboratory tests are neither highly sensitive nor specific for infection as a cause of non-union. The gold standard proof of infection is from histological examination and pathogen-positive cell cultures of the infected area, which requires invasive biopsy. Non-invasive diagnostic tests include plain radiography, CT, ultrasonography, infection scintigraphy, and MRI. It is the last approach that may be the first course of imaging work-up of a suspected infection [5]. MRI can exclude osteomyelitis with high certainty [6]. However, it cannot distinguish with the same certainty between osteomyelitis and noninfectious causes of abnormal marrow signal intensity, such as neuropathic arthropathy and reactive marrow edema [6]. Furthermore, MRI can be hampered by the presence of screws, plates, or other metallic devices at the site of bone injury [7].

In a previously published study, [^18^F]FDG PET demonstrated high sensitivity in identifying the presence of osteomyelitis in orthopedic surgery patients with nonspecific clinical symptoms of infection [8]. In a meta-analysis of studies on suspected osteomyelitis, PET/CT with [^18^F]FDG has shown promising sensitivity, specificity, and accuracy in comparison to bone scintigraphy or leukocyte scintigraphy [9]. Despite these findings, there has hitherto been no study systematically evaluating [^18^F]FDG PET for diagnosis of suspected infections at non-union fractures. The aim of our study, therefore, was to investigate whether increased [^18^F]FDG accumulation in non-union fracture sites affords a non-invasive imaging biomarker for infections in cases confirmed by intraoperative tissue culture or clinical follow-up.

## Methods

### Patient population

This study was approved by the institutional ethics committee.

From a large PET database at the University of Munich, we retrospectively identified 35 patients with suspected infection of non-union fractures who had been treated in a level I trauma center. The patients had undergone either [^18^F]FDG PET/CT (*N* = 24), [^18^F]FDG PET (*N* = 11) with additional CT (*N* = 8), or conventional X-ray (*N* = 3) between 2000 and 2015.

Patients presented with persistent pain at the fracture site and may have also reported abnormal movement or clicking at the level of the fracture. The geneses of the non-unions were trauma, wedge osteotomy, osseous segment transport, and arthrodesis or fatigue fractures. Clear clinical markers for acute local infection such as fistulas or pyrophoric wounds, erythema, and/or hyperthermia were absent. Laboratory findings (CRP, leukocytes), risk factors (atherosclerosis, alcohol, adiposity, vascular disease, history of infection, smoking or nicotine use, neurology, gout, and renal insufficiency), a surgically implanted metallic device, gender, age, body mass index (BMI), and location of injury were recorded. At the time of the [^18^F]FDG PET scan, patients were not being treated with antibiotics.

### [^18^F]FDG PET and PET/CT scan

Partial-body PET scans were acquired in three-dimensional (3D) mode using different PET scanners (*N* = 5 GE Discovery 690 CT, *N* = 11 ECAT PET scanner, *N* = 14 Philips Gemini CT, *N* = 5 Siemens BioGraph 64). Among the 35 patients, 24 had undergone an [^18^F]FDG PET/CT, and 11 had undergone only standalone [^18^F]FDG PET. Based on the National Electrical Manufacturers Association (NEMA) NU2-2001 standard, phantom studies were conducted, and standardized uptake value (SUV) conversion factors were calculated to allow valid pooling of the results. After patients had fasted for at least 6 h and blood glucose levels were measured to exclude cases of blood glucose levels above 150 mg/dL (mean 97 ± 10 ng/mL prior to scan), a diuretic was administered intravenously (furosemid, Furorese 20 mg; Hexal AG, Holzkirchen, Germany), closely followed by bolus administration of [^18^F]FDG (mean 254 ± 72 MBq) according to body mass. The emission recording sequence was initiated 60 min after intravenous injection of [^18^F]FDG. Attenuation correction was based on either CT or an external rotating ^68^Ge source. CT was performed as high-dose CT (automated dose modulation, mean 220 mAs; 120 kV, CT slice 3 mm) with (*N* = 20) or without contrast agent (*N* = 4). When contrast agent was administered prior to the CT scan, it consisted of a mean weight-adapted volume of 120 mL iodine-containing contrast agent (iomeprol, Imeron^®^ 350 mg iodine/mL, Bracco Imaging Deutschland GmbH, Konstanz, Germany), administered intravenously at a rate of 2.5 mL/s; the CT scan was initiated 50 s after the beginning of the infusion of contrast agent in order to depict the venous contrast medium phase. A dedicated software package was used for image reading (Hermes Hybrid Viewer, version 2.0; Hermes Medical Solutions, Stockholm, Sweden). In the present retrospective study, the images were interpreted by consensus of two experienced nuclear medicine physicians and a radiologist, who were blinded to bacteriological and surgical data and the patients’ clinical follow-up.

### Image interpretation

For image interpretation, we performed quantitative SUV-based analysis, visual analysis, and a combination of both assessments.

1. **SUV-based analysis**
  Within the SUV based analysis, we performed further receiver operating characteristic (ROC) analysis to obtain a cut-off value. Consequently, all patients with SUV_max_ greater than the cut-off value were rated positive, and those with SUV_max_ less than the cut off-value were rated negative for infection.
2. **Visual analysis**
  We interpreted images positive for infection of the non-union using two different criteria.

- First set: The first qualitative set considered the mere presence of [^18^F]FDG activity—whether homogenous or focal—at the bone–bone interface or the prosthesis–bone interface as indicative of infection, regardless of intensity.
- Second set: On the basis of two previously published approaches [10, 11], we defined infection as follows:
  Positive for infection:
  - [^18^F]FDG uptake was asymmetric compared to that in the contralateral extremity, and
  - [^18^F]FDG uptake was focally accentuated at the bone–bone, bone–implant, or bone–soft tissue interface, thus constituting a distinct “hotspot”, and
  - [^18^F]FDG uptake detected along the course of the non-union fracture was at least twice the mean uptake of inactive muscle from the contralateral extremity (SUV_max_ [fracture] > 2 x SUV_mean_ [muscle]).
  Negative for infection:
  - little [^18^F]FDG uptake, and no excessive increase in uptake;
  - in the case of weakly elevated [^18^F]FDG uptake, the uptake pattern was smooth and evenly distributed along the entire surface of the fracture line or along the surface of the prosthesis;
  - no distinct foci were discernible;
  - a weak [18F]FDG uptake at the end of metallic devices, where movement is mechanically constrained, and a weak [^18^F]FDG uptake which could be unequivocally attributed to the part of the bone experiencing the highest mechanical stress, e.g. weight-loading.

3. **Combination of visual diagnosis and SUV-based diagnosis**
  In addition to these analyses, we performed a combination of the second set of visual analysis and the SUV-based diagnosis. In this approach, [^18^F]FDG PET was rated as positive only if visual assessment and SUV-based analysis were concordant.

### Results validation

PET findings were verified by intraoperative tissue cultures (*N* = 25) or long term clinical follow-up for more than one year (*N* = 10). The clinical assessment and other follow-up data were registered during regular visits to the trauma center.

True positive (TP), true negative (TN), false positive (FP), and false negative (FN) were defined as follows:

- TP:
  - A positive PET result according to the above definitions, and
  - positive intraoperative bone microbiology or, in the case of follow-up, a radiologically and clinically eventful follow-up with the occurrence of clinical symptoms such as pain at the infection site, drainage from the area, fever, tenderness, redness, and warmth or swelling around the affected bone, lost range of motion, or increasing laboratory parameters for infection (leukocytes, CRP level);
- TN:
  - negative PET result according to our given definitions, and
  - negative intraoperative bone microbiology or, in the case of follow-up, a radiologically and clinically uneventful follow-up without clinical symptoms, and with normal infection parameters with no further therapy for at least one year;
- FP:
  - positive PET result according to our given definitions, and
  - negative intraoperative bone microbiology or negative follow-up;
- FN:
  - negative PET result according to our given definitions, and
  - positive intraoperative bone microbiology or negative follow-up.

### Statistical analysis

Statistical analyses were performed using the SPSS statistical software package (version 22.0; IBM Corp., Armonk, NY, USA). A *p* value of less than 0.05 was considered statistically significant. Comparisons of variables between clinically infected and non-infected groups were performed using the *t* test for parametric data, the chi-square test for ordinal data, and the Mann–Whitney *U* test for nonparametric data. Sensitivity, specificity, positive predictive value (PPV), and negative predictive value (NPV) were calculated by comparing the PET results to intraoperative findings or clinical follow-up.

## Results

### Patient cohort

A general overview of clinical and patient data is given in Table 1.

**Table 1:** Patient characteristics for patients with or without evidence of clinical infection (*P* = NS for all comparisons between groups)

		Evidence of clinical infection *N* = 13	No evidence of clinical infection *N* = 22	Total *N* = 35
Age (years)	(mean ± SD)	53 ± 11	47 ± 5	50 ± 15
Gender	Female	3	3	6
	Male	10	19	29
Infection proven by	Intraoperative microbial bone culture	12	13	25
	Follow-up > 1 year	1	9	10
CRP (mg/dL)	(mean ± SD)	0.4 ± 0.2	0.5 ± 0.4	0.5 ± 0.3
Leukocytes (/nl)	(mean ± SD)	8.0 ± 2.3	6.8 ± 1.6	7.2 ± 1.9
BMI (kg/m^2^)	(mean ± SD)	27.2 ± 4.0	27.9 ± 5.1	27.6 ± 4.6
Location of non-union	Femur	3	4	7
	Tibia	7	10	17
	Fibula	0	4	4
	Tibia and fibula	1	1	2
	Foot	1	2	3
	Humerus	1	0	1
	Patella	0	1	1
Risk factors	None	2 (15 %)	3 (14 %)	5 (14 %)
	Single	7 (54 %)	8 (36 %)	15 (43 %)
	Multiple	4 (31 %)	11 (50 %)	15 (43 %)
	Adiposity	4	7	11
	History of infection	1	8	9
	Nicotine	5	6	11
	Alcohol	4	3	7
	Vascular disease	2	3	5
	Gout	0	2	2
	Neurological symptoms	0	3	3
	Diabetes	0	2	2
	Renal insufficiency	1	0	1
	COPD	1	0	1
Metallic implants	Yes	6	6	12
	No	7	16	23
Radiological classification	Atrophic	5	6	11
	Oligotrophic	8	10	18
	Hypertrophic pseudarthrosis	0	6	6
Persistence of non-union (months)	(mean ± SD)	11.2 ± 6.1	16.9 ± 18.8	14.8 ± 15.4
PET/CT technique	CT with contrast	7	13	20
	CT without contrast	1	3	4
	PET only	5	6	11

*NS* non-significant, *CRP* C-reactive protein, *BMI* body mass index, *COPD* chronic obstructive pulmonary disease

In 13 of the 35 patients (37 %), infection was proven either by positive intraoperative tissue culture (*N* = 12) or by positive follow-up (*N* = 1). Infections were caused by a single gram-positive pathogen in nine patients (*Streptococcus epidermidis*, *N* = 3; *Streptococcus aureus*, *N* = 3; *Enterococcus faecalis*, *N* = 1; *Staphylococcus capitis* *N* = 2) and by mixed infections in three patients (*Streptococcus epidermidis*, *Streptococcus aureus*, *group A ß-hemolytic streptococcus*, *Pseudomonas*, and *Escherichia coli*). In the remaining 22 patients (63 %), there was no evidence of infection. In 12 patients (38 %), the [^18^F]FDG PET scan was performed in the presence of metallic implants. Specifically, seven patients had been treated with plate osteosynthesis, two patients with intramedullary nail, and one patient each by screws, by external fixation along with inserted screws, and with combined plate osteosynthesis of the fibula and intramedullary nail placement in the tibia. Non-union types were classified as oligotrophic (*N* = 18), atrophic (*N* = 11), and hypertrophic pseudarthrosis (*N* = 6) based on radiological criteria. The mean time of persistence of the non-unions was 14.8 (±15.4) months. There were no statistical differences in patient characteristics between the clinically infected and non-infected groups (Table 1).

### Results of [^18^F]FDG PET

The association of [^18^F]FDG-PET findings and laboratory findings, risk factors, BMI and radiological classification are listed in Table 2. Patient classification according to type of non-union are shown in Table 3. The results for the SUV-based, visual, and combined analysis are presented in Table 4. Within the SUV-based analysis, a cut-off value of 4.3 was obtained by ROC analysis (AUC 0.848, sensitivity 75 %, specificity 77 %).

**Table 2:** [^18^F]FDG PET findings* according to normal or abnormal laboratory findings, risk factors, BMI, and radiological classification of non-unions (*P* = NS for all comparisons between groups)

		PET positive for infection (*N* = 14)	PET negative for infection (*N* = 21)	Total (*N* = 35)
CRP level (*N* = 31)	<0.3 mg/dL	5	9	14
	≥0.3 mg/dL	7	10	17
Leukocytes (*N* = 31)	4.3 to 10.8/nl	11	19	30
	≥10.8/nl	1	0	1
Risk factors (*N* = 35)	None	3	2	5
	Single	6	9	15
	Multiple	5	10	15
BMI (kg/m^2^) (*N* = 34)	18.5 to <25 kg/m^2^	6	4	10
	≥25 to <30 kg/m^2^	6	8	14
	≥30 kg/m^2^	2	8	10
Type of non-union (*N* = 35)	Atrophic	5	6	11
	Oligotrophic	8	10	18
	Hypertrophic	1	5	6

Normal values: CRP <0.3 mg/dL; leukocytes 4.3–10.8/nl

*Based on the second set of visual analysis

*BMI* body mass index, *NS* non-significant

**Table 3:** Classification of patients according to type of non-union*

Types of non-union	No.	TP	TN	FP	FN
Atrophic	11	4 (36 %)	5 (45 %)	1 (9 %)	1 (9 %)
Oligotrophic	18	7 (39 %)	9 (50 %)	1 (6 %)	1 (6 %)
Hypertrophic	6	0 (0 %)	5 (83 %)	1 (17 %)	0 (0 %)

*Based on the second set of visual analysis

*TP* True positive, *TN* true negative, *FP* false positive, *FN* false negative

**Table 4:** Results of different analysis criteria

	Quantitative analysis	First set of visual analysis	Second set of visual analysis	Combination of quantitative and second set of visual analysis
TP	9	11	11	8
TN	17	9	19	20
FP	5	14	3	2
FN	4	1	2	5
Sensitivity	69 %	91 %	85 %	62 %
Specificity	77 %	39 %	86 %	91 %
PPV	64 %	44 %	79 %	80 %
NPV	81 %	90 %	90 %	80 %
Accuracy	74 %	57 %	86 %	80 %

*TP* True positive, *TN* true negative, *FP* false positive, *FN* false negative, *PPV* positive predictive value, *NPV* negative predictive value

**Fig. 1:**
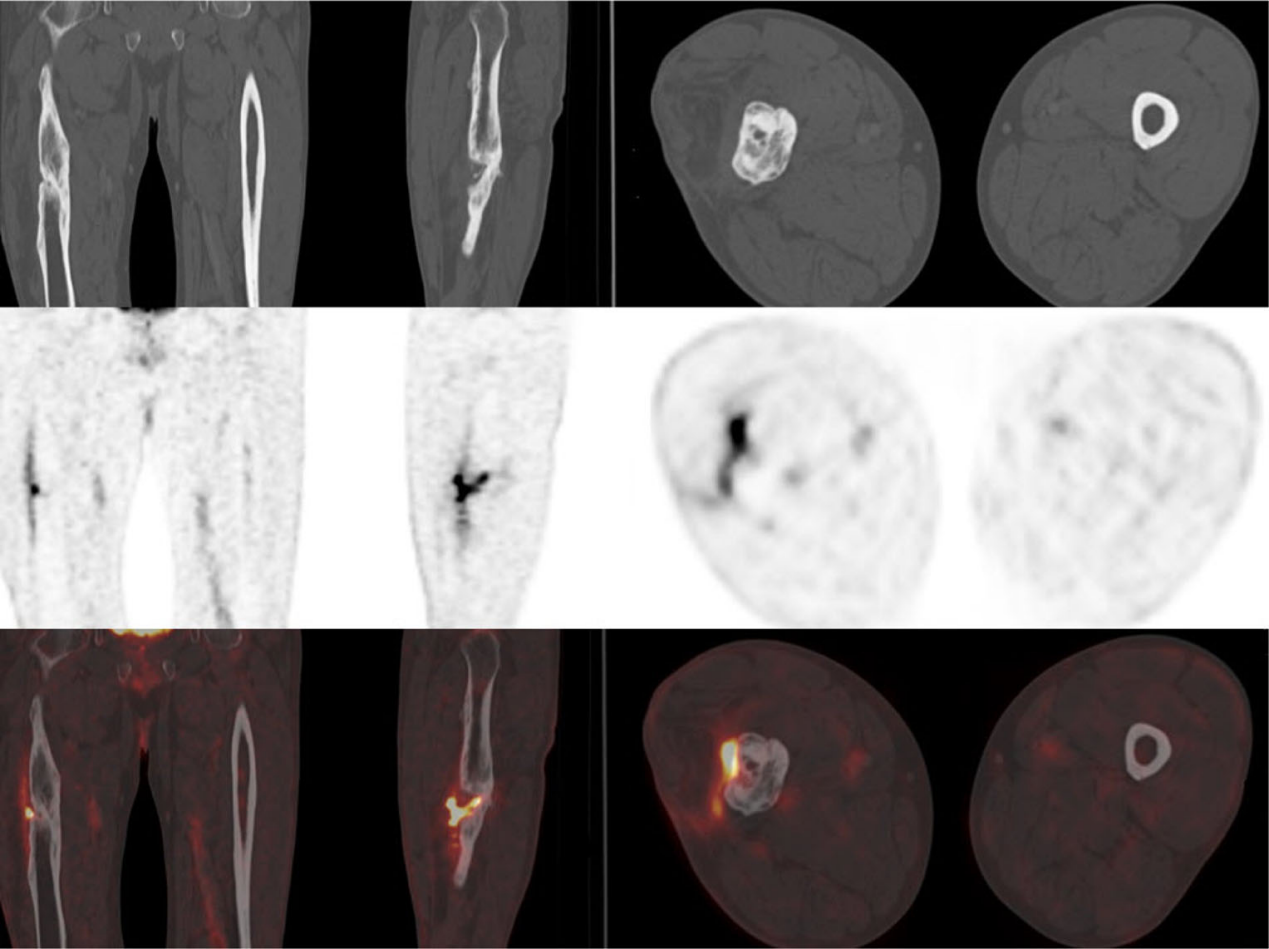
55-year-old patient with oligotrophic non-union. No complete osseous consolidation was seen for 6 months. Pathological, irregularly distributed [^18^F]FDG uptake (SUV_max_ 7.7, SUV_ratio_ 7.4) was found along the bone–bone interface, with a clear “hotspot” along the lateral fissure of the femur. The elevated [^18^F]FDG uptake extends to the proximate soft tissue. Atrophy of the *M. vastus lateralis* is seen due to immobilization. The [^18^F]FDG PET/CT scan was rated true positive in all image analysis based on positive intraoperative microbial tissue culture

**Fig. 2:**
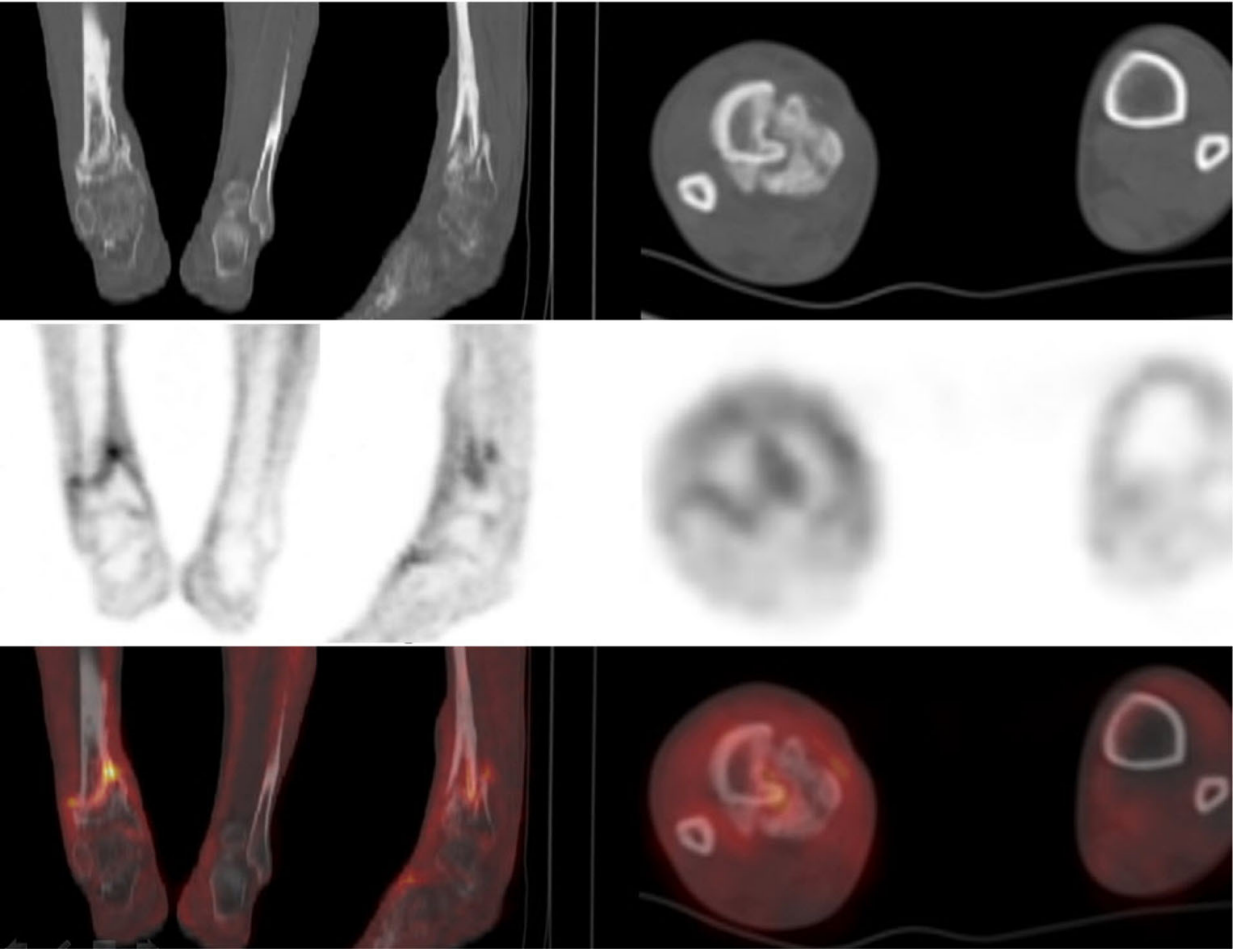
44-year-old patient with oligotrophic non-union fracture of the distal tibia. No complete osseous consolidation was seen for 16 months. [^18^F]FDG uptake was seen along the bone–bone interface of the distal tibia. The [^18^F]FDG uptake was focally increased (SUV_max_ 4.2, SUV_ratio_ 3.1) at the distal part of the proximal bone segment. Only the first and second sets of visual analysis were interpreted as infection of the non-union (true positive). The intraoperative tissue culture was positive

**Fig 3:**
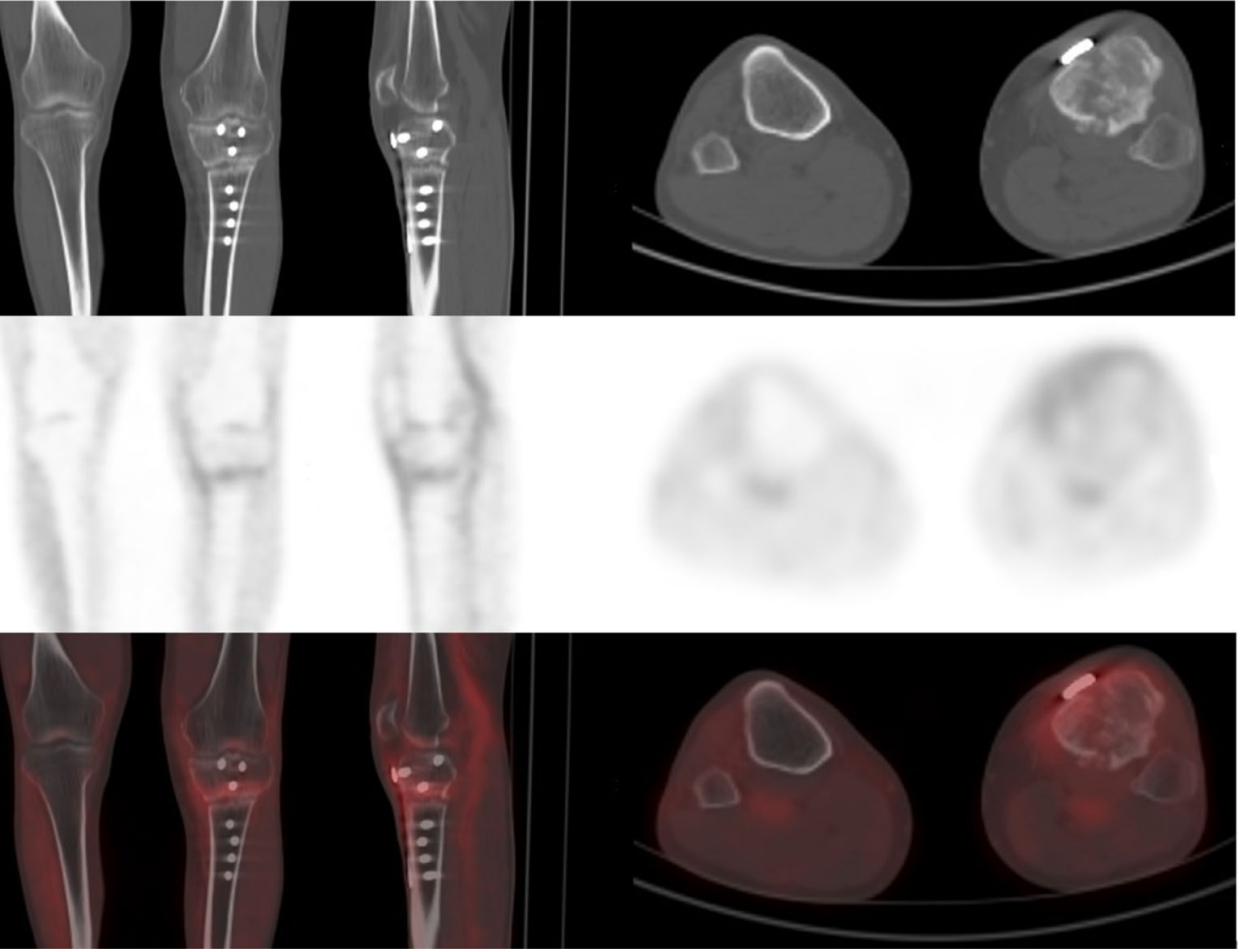
53-year-old patient with atrophic non-union fracture after a tibial wedge osteotomy. No complete osseous consolidation was seen for 9 months. [^18^F]FDG PET scan showed only slight, homogeneously distributed and not focally accentuated uptake (SUV_max_ 3.0, SUV ratio 1.8) along the fracture gap, which was fixed by a plate. The uptake was rated positive for infection in the first set of visual analysis (false positive). The SUV-based analysis, the second set of visual analysis, and the combination of both were rated negative for infection (true negative). In the follow-up, no infection was observed

The best results were achieved by implementing the second set of visual analysis: [^18^F]FDG PET was rated positive for infection in 14 of 35 patients (40 %). There were 11 TP, 19 TN, three FP, and two FN results, indicating 85 % sensitivity, 86 % specificity, 79 % PPV, 90 % NPV, and 86 % accuracy. The [^18^F]FDG accumulation in infected non-unions was more circumscribed than that in non-infected non-unions, and typically did not affect the entire surface of the fracture line. In non-infected non-unions, we saw more homogenous [^18^F]FDG uptake at the entire surface of the fracture line (Figs. 1, 2, and 3). Furthermore, the uptake of infected non-unions was significantly higher. The SUV_max_ (6.4 ± 2.7) in the clinically infected non-union group was significantly higher than that in the non-infected group (3.0 ± 1.7; *p* < 0.01). Similarly, the mean SUV_ratio_ in the infected group (5.3 ± 3.3) was higher than that in the non-infected group (2.6 ± 1.5; *p*<0.01).

Applying the second set of visual analysis [^18^F]FDG PET revealed four TP, five TN, one FP, and one FN case among the atrophic non-unions; seven TP, nine TN, one FP, and one FN for the oligotrophic non-unions; and five TN and one FP case among the instances of hypertrophic non-unions (Table 3).

## Discussion

Infection at the site of a fraction can delay union, or can result in a non-union, which is a potentially disabling persistent failure of fracture healing. By definition, a non-union will not heal unless properly treated in accordance with the cause of the failure. It is of the utmost importance, therefore, to rule out suspected infection in cases of non-union fractures so as to facilitate timely and appropriate surgical fixation treatments. While several imaging modalities have found use in the investigation of potentially infected non-union fractures, [^18^F]FDG PET has not hitherto been tested. We thus present the first systematic evaluation of [^18^F]FDG PET exclusively in patients with non-union fractures and suspected infections.

We tested several approaches for evaluating the imaging results. Applying the second set of the visual analysis, [^18^F]FDG PET showed high diagnostic sensitivity of 85 % and specificity of 86 %, which should afford a secure diagnosis of infected non-union fractures. Furthermore, the high NPV of 90 % should help to avoid inappropriate attempts to treat with antibiotics or surgical debridement when infection is absent. Thus, we demonstrate that [^18^F]FDG PET reliably differentiates infected and non-infected non-unions, and should then be a useful adjunct for therapeutic management.

Metallic implants are vulnerable sites of postoperative infection; in a previously published [^18^F]FDG PET study, a subgroup of six of 22 patients with suspected metallic implant-associated infections had non-union fractures. These six patients were rated as having TN findings [12], indicating that [^18^F]FDG uptake at uninfected non-union sites is significantly lower than that at the sites of actual infections. Another study evaluated [^18^F]FDG PET/CT in trauma patients with suspected chronic osteomyelitis [13]; the authors found that six of 33 patients with suspected osteomyelitis had non-union fractures, of which three were rated true positive, two as true negative, and one as false positive, due to pronounced [^18^F]FDG uptake within the area of the fracture, despite absence of infection. Our investigation in a larger series confirms these preliminary published results [12, 13].

In the absence of infection, [^18^F]FDG accumulation may be elevated in non-union fractures due to imperfect fixation, with sterile inflammation arising from the resultant friction. However, we expected that the [^18^F]FDG uptake of the infected non-union should significantly exceed that seen with non-infected non-union. Indeed, clear discernment of the distinction between aseptic inflammation and infection did not pose a problem in the present study.

A standardized approach to the interpretation of [^18^F]FDG PET for diagnosing infection in non-union fractures is presently lacking. In our study, no reliable SUV-based threshold for more accurate diagnosis in individual patients emerged. Likewise, it has been reported that there is no simple relation between the probability of infection and the intensity of periprosthetic activity [14]. Considering merely the presence of activity at the bone–bone interface or the prosthesis–bone interface cannot be unambiguously associated with infection. These results are in line with previously published studies addressing periprosthetic [^18^F]FDG uptake [10, 15, 16]. We find that the particular pattern of pathological uptake must be considered. Applying the second set of visual analysis resulted in achieving high sensitivity of 85 % and specificity of 86 %. [^18^F]FDG uptake at the infection sites was focally increased, and typically did not affect the entire surface of the non-union fracture. Locally increased stress and local friction can also lead to focally circumscribed uptake, which is generally less pronounced than that due to infection. In non-infected non-unions, [^18^F]FDG uptake was more homogenous along the fracture line. These patterns are likely due to aseptic inflammation provoked by persistent movement and friction along the entire surface of the non-union fraction. Regarding the pathological pattern in this visual analysis, the number of false-positive findings was lower than in the SUV-based quantitative analysis. We thus concur with the findings of Chacko et al. [16], who noted that the intensity of [^18^F]FDG uptake is less informative than is the location of the increased signal. The combination of the visual and quantitative analysis seems to underperform the proposed visual method, because no diagnostically reliable SUV_max_ cut-off value could be identified.

Hypertrophic, oligotrophic, and atrophic radiographic appearance allows estimation of the degree of fracture stability and also the biologic viability of the fracture fragments. It is likely that different forms of fracture non-unions may be responsible for the differences in [^18^F]FDG uptake patterns. In particular, we suppose that hypertrophic non-unions may have a higher physiological [^18^F]FDG uptake than oligotrophic or atrophic non-unions, due to more distinct bone remodeling. In our study, hypertrophic non-union was radiologically diagnosed in six patients, of whom five were rated as true negative and one as false positive. In that individual, hypertrophy might have aggravated the diagnosis. However, the other five patients with hypertrophic non-union were rated correctly, thus indicating that hypertrophy is not a contraindication for the [^18^F]FDG PET method. Further studies are needed to examine the diagnostic accuracy in the relatively rare patients with hypertrophic non-union and suspected infections.

Contemporary nuclear medicine offers several imaging modalities for use in diagnosing infection in bone. At present, white blood cell (WBC)/bone marrow imaging is considered the best functional diagnostic imaging test for infected prosthetic joints [14, 17]. The diagnostic value of combined imaging with ^111^In-labeled leukocytes and ^99m^Tc methylene diphosphonate for the diagnosis of non-union fractures was discussed in an earlier study [18], with the authors reporting sensitivity, specificity, PPV, NPV, and accuracy of 86 %, 84 %, 69 %, 94 %, and 82 %, respectively. Except for the lower PPV, these results seem comparable to those from our second set of [^18^F]FDG PET visual analysis, which may call into question the additional value of single-photon emission computerized tomography (SPECT)/CT in WBC in patients with suspicion of infection at non-union fractures. Thus far, no multimodal imaging comparison has been performed regarding diagnostic accuracy in patients with non-unions, and the best imaging modality for non-union fractures remains to be identified. Images obtained with PET have much higher resolution than scintigraphy images [19], and [^18^F]FDG PET has an inherently high target-to-background ratio. Furthermore, the procedure is widely available. Diagnostic results from [^18^F]FDG PET can be secured within 2 h, which is favorable for patient welfare. Our results indicate that [^18^F]FDG PET/CT or [^18^F]FDG PET with additional morphological imaging represents a plausible alternative for imaging infections of non-unions.

## Limitations

Although this retrospective study dealt with a larger group than previously published works, the total number of patients was relatively small. Thus, larger series of patients will be needed to confirm our results. Unfortunately, we were not able to perform a multi-modality imaging comparison of diagnostic accuracy. However, it will eventually be important to ascertain the diagnostic accuracy of MRI, [^18^F]FDG PET/CT, and infection scintigraphy for the detection of infected non-union fractures. Of note, our sensitivity and specificity values were assessed in a homogeneous group of patients who were not receiving any antibiotic medication at the time of the [^18^F]FDG PET scan. Whether current antibiotic treatment might impair sensitivity in the PET/PET-CT investigation needs to be evaluated in a separate study. A major drawback of [^18^F]FDG PET is the nonspecific uptake that may occur adjacent to prostheses, in healing tissues, bone fractures, varicose veins, and atherosclerotic lesions [20–22].

## Conclusion

[^18^F]FDG PET differentiates infected and non-infected non-unions with high accuracy in patients for whom clinical findings are inconclusive for a local infection. This approach should be considered for therapeutic management of non-unions.
